# A multimodal regional intervention strategy framed as friendly competition to improve hand hygiene compliance

**DOI:** 10.1017/ice.2018.261

**Published:** 2019-02

**Authors:** Manon D. van Dijk, Sanne A. Mulder, Vicki Erasmus, A. H. Elise van Beeck, Joke M. J. J. Vermeeren, Xiaona Liu, Ed F. van Beeck, Margreet C. Vos

**Affiliations:** 1 Department of Medical Microbiology and Infectious Diseases, Erasmus MC, University Medical Center Rotterdam, Rotterdam, The Netherlands; 2 Department of Public Health, Erasmus MC, University Medical Center Rotterdam, Rotterdam, The Netherlands; 3 Department of Quality and Patient Care, Erasmus MC, University Medical Center Rotterdam, Rotterdam, The Netherlands

## Abstract

**Objective:**

To investigate the effects of friendly competition on hand hygiene compliance as part of a multimodal intervention program.

**Design:**

Prospective observational study in which the primary outcome was hand hygiene compliance. Differences were analyzed using the Pearson χ^2^ test. Odds ratios (ORs) with 95% confidence interval were calculated using multilevel logistic regression.

**Setting:**

Observations were performed in 9 public hospitals and 1 rehabilitation center in Rotterdam, Netherlands.

**Participants:**

From 2014 to 2016, at 5 time points (at 6-month intervals) in 120 hospital wards, 20,286 hand hygiene opportunities were observed among physicians, nurses, and other healthcare workers (HCWs).

**Intervention:**

The multimodal, friendly competition intervention consisted of mandatory interventions: monitoring and feedback of hand hygiene compliance and optional interventions (ie, e-learning, kick-off workshop, observer training, and team training). Hand hygiene opportunities, as formulated by the World Health Organization (WHO), were unobtrusively observed at 5 time points by trained observers. Compliance data were presented to the healthcare organizations as a ranking.

**Results:**

The overall mean hand hygiene compliance at time point 1 was 42.9% (95% confidence interval [CI], 41.4–44.4), which increased to 51.4% (95% CI, 49.8–53.0) at time point 5 (*P*<.001). Nurses showed a significant improvement between time points 1 and 5 (*P*<.001), whereas the compliance of physicians and other HCWs remained unchanged. In the multilevel logistic regressions, time points, type of ward, and type of HCW showed a significant association with compliance.

**Conclusion:**

Between the start and the end of the multimodal intervention program in a friendly competition setting, overall hand hygiene compliance increased significantly.

According to the World Health Organization (WHO), adverse events in healthcare are a growing problem worldwide, and healthcare-associated infections (HAIs) are among the most frequent events.^1^ An HAI is an infection that a patient acquires during hospitalization, which increases the risk of morbidity, mortality, and a prolonged hospital stay.^2^ ^–^ ^4^ In European acute-care hospitals, the prevalence of HAI was 6.0% in 2012.^5^ Furthermore, the overall cost of HAIs per year is US$8 billion in Europe and US$4.5 billion in the United States.^1^ ^,^ ^6^

The prevalence of HAI is strongly associated with the type of medical service provided and the behavior of healthcare workers (HCWs).^7^ This includes the hand hygiene behavior of HCWs. The contaminated hands of HCWs have been identified as the cause of several outbreaks.^8^ ^,^ ^9^ An effective and recommended solution to prevent transmission of microorganisms is improving hand hygiene compliance in healthcare organizations. Hand hygiene can consist of (1) hand washing with water and soap followed by drying the hands with paper towels or (2) hand disinfection with an alcohol-based hand rub (ABHR). HCWs in the Netherlands are required to follow the guidelines for hand hygiene as provided by the WHO.^10^ Adherence to these guidelines is monitored by infection prevention professionals within organizations, as well as the national health inspectorate. The WHO refers to Five Moments of Hand Hygiene: (1) before touching a patient, (2) before a clean procedure, (3) after body fluid exposure, (4) after touching a patient, and (5) after touching a patients’ environment.^11^

Despite clear guidelines and monitoring, hand hygiene compliance among HCWs in healthcare organizations is unacceptably low.^12^ Furthermore, research shows that, among others, the type of ward and the type of HCW (eg, nurses, physicians, or other) in organizations are determinants for hand hygiene compliance.^13^ ^–^ ^16^ Studies that have investigated hand hygiene compliance among different types of HCWs have found that compliance is higher among nurses than physicians and other HCWs.^17^

Multiple intervention studies have been performed to increase hand hygiene compliance among HCWs.^18^ ^–^ ^20^ These studies suggest that different types of interventions could increase hand hygiene compliance in healthcare organizations. Despite these efforts, it remains challenging to sustain long-term effects.^21^ ^,^ ^22^

To date, few studies have included friendly competition within and between hospitals in their intervention designs to encourage HCWs to improve hand hygiene.^21^ For example, Salman et al^22^ implemented a motivation scoreboard with hand hygiene compliance results of HCWs to improve the compliance rate. Gaynor et al^23^ examined the impact of competition between hospitals on efficiency and concluded that length of stay decreased more for hospitals in a competitive market.^24^

In conclusion, interventions to increase hand hygiene compliance in healthcare organizations are an important but complex topic. To our knowledge, interventions in a natural setting with the involvement of different healthcare organizations in friendly competition are rare. We aimed to investigate the effects of friendly competition on hand hygiene compliance by implementing a WHO multimodal regional intervention strategy framed as a friendly competition intervention program. The second objective was to gain insight into the differences in hand hygiene compliance between wards and types of HCWs.

## Methods

### Study design

In 2014, the “Collaborating Rijnmond Hospitals” program “Roll Up Your Sleeves” started in 10 healthcare organizations (9 hospitals and 1 rehabilitation center) in the greater Rotterdam region. During this prospective comparative observational study, the effect of friendly competition (between and within the healthcare organizations) on hand hygiene compliance was observed as the primary outcome. Compliance with hand hygiene was observed on different wards and among different HCWs within each healthcare organization at 5 time points between May 2014 and September 2016.

### Study population

**Fig. 1:**
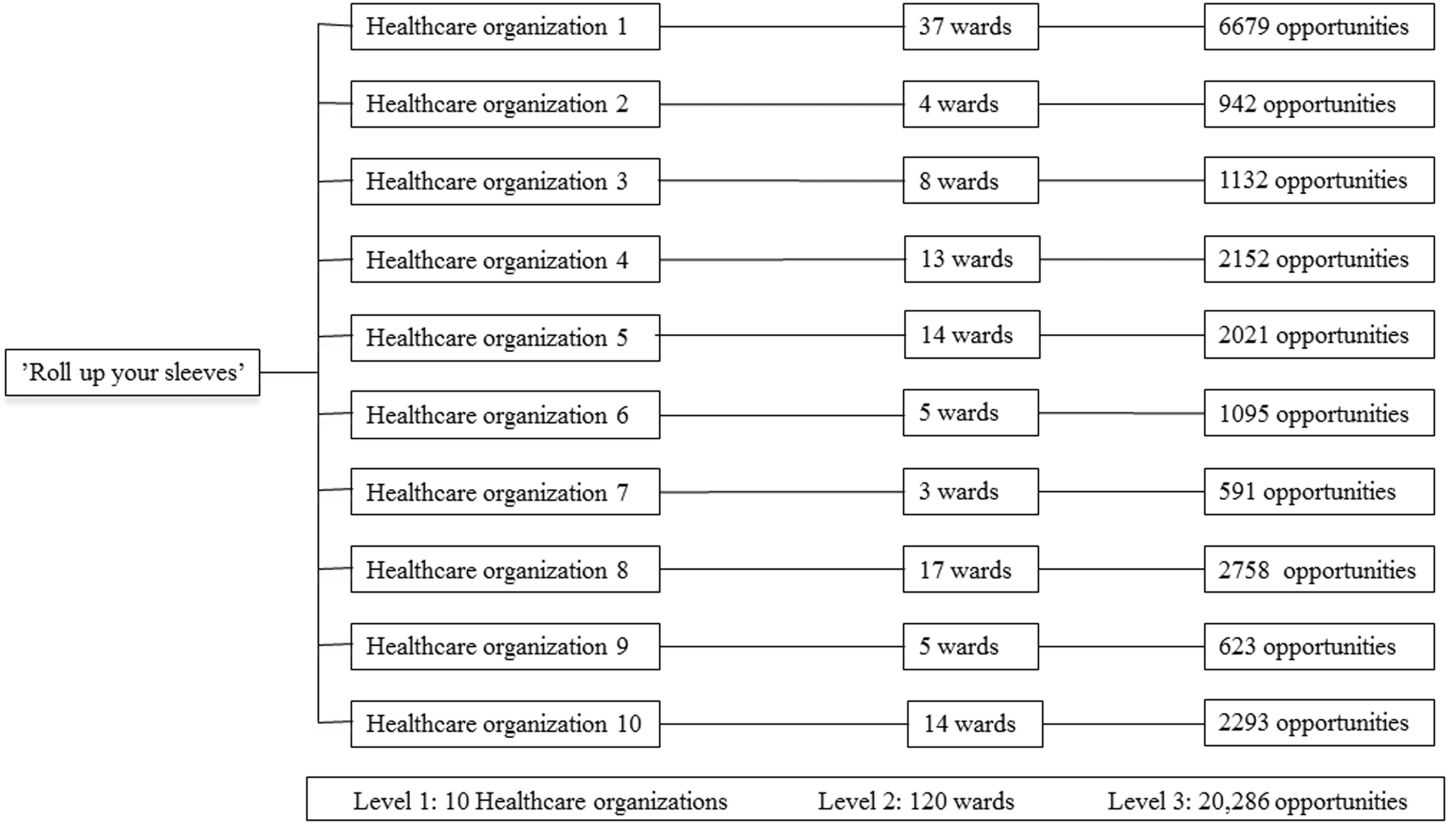
Overview of the study population, which illustrates a hierarchical overview with 3 levels: healthcare organizations, wards, and observed opportunities. Level 3 illustrates the number of observed opportunities that hand hygiene should have been applied.

The “Roll Up Your Sleeves” project was implemented in 10 healthcare organizations. Only HCWs that had physical contact with patients were included in the observations. In total, 20,286 hand hygiene opportunities were observed on 120 wards. Figure 1 shows a hierarchical overview of the 3 levels in the study: the organizational level, the ward level, and observed hand hygiene opportunities.

### Intervention

The “Roll Up Your Sleeves” intervention was based on monitoring and feedback of achievement at 6-month intervals over 2 years. In addition, multiple (optional) training elements were offered. (1) Individual e-learning was provided about hand hygiene techniques and opportunities, different for nurses and physicians. (2) A kick-off workshop was conducted in which stakeholders (primarily infection control staff, ward managers, or head nurses) from each organization defined the implementation strategy for their organization by setting up a framework with rules, priorities, and goals regarding hand hygiene. (3) Team training was offered in a train-the-trainer setting; several stakeholders (usually infection control staff and ward nurses) from each organization were trained to train other HCWs in their own organization on how to improve hand hygiene team performance on wards. (4) In observer training, infection prevention specialists and HCWs were trained to perform their own internal audits. All interventions were delivered by an external trainer of the “Collaborating Rijnmond Hospitals” program, and the observer training was based on the “Hand Hygiene Australia” protocol.^11^ As a fifth (and compulsory) core element of the program, the stakeholders of each organization received the standardized feedback report on hand hygiene compliance after each round of observations. In total, 5 observation rounds were conducted over a 2-year period at 6-month intervals. At the director level, the results were presented during annual meetings of the hospital collaborative to which all institutions belonged. Furthermore, the program leader within each organization (usually an infection control practitioner) received a feedback report and was responsible for distributing the results within the organization, usually via newsletters, ward reports or hospital websites. This report included the hand hygiene compliance rate on the organization level (aggregated) and the ward level. During an annual conference, the overall results were presented and the organizations and wards with best results received a prize. The added value of this friendly competition setting is that healthcare organizations can learn from each other and keep challenging themselves to improve.

### Data collection

HCWs were unobtrusively observed by trained observers at 5 time points (at 6-month intervals) from May 2014 to September 2016. New independent (medicine) students and research assistants were trained for each round of observations. The students had to participate in a training by an external trainer of the “Collaborating Rijnmond Hospitals” program. At the end of the training, observers were tested on their knowledge and observation skills using an auditor test developed by Hand Hygiene Australia. For the observations, the Hand Hygiene Australia observation instrument was used. This instrument is based on the Five Moments of Hand Hygiene described by the World Health Organization (WHO).^10^ ^,^ ^11^ Data were collected during a 2-hour period (8:00–10:00 a.m.), and at least 3 nurses were followed and observed. Physicians and other HCWs who assisted the observed nurses were also included in the observations.

### Statistical analysis

The dependent variable in this study was hand hygiene compliance, calculated by dividing the number of correct hand hygiene opportunities by the total number of hand hygiene opportunities. The independent variables in this study were the 5 time points, different ward types, and type of HCW (ie, physician, nurse, or other). Hand hygiene compliance was coded as “missed” or “rub/washed.”

A χ^2^ analysis was performed to investigate the association between the dependent variable and each individual independent variable, comparing time point 1 with 5. Univariable and multivariable multilevel logistic regressions were performed to investigate the association between hand hygiene compliance and different time points, ward types, and type of HCW. The outcome variable of ward type and type of HCW in the multilevel analyses was the average compliance of all 5 time points for all 10 healthcare organizations.

Associations were considered statistically significant at *P*<.20 for univariable analyses and *P*<.05 for multivariable analyses. Furthermore, the outcome resulted in odds ratios (OR) with a corresponding confidence interval (95% CI). All data were analyzed using SPSS software version 21 or 24 (IBM, Armonk, NY).

## Results

### Hand hygiene compliance

**Fig. 2:**
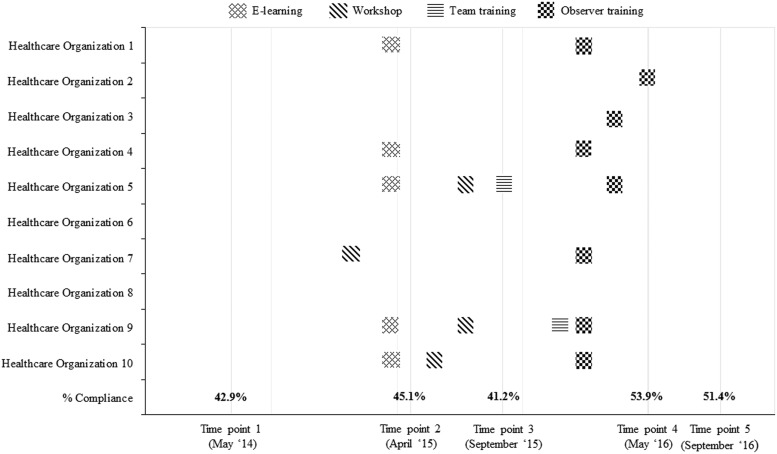
Overview of the mean hand hygiene compliance per time point of the 10 healthcare organizations in combination with the implemented (optional) interventions.

Figure 2 shows the mean hand hygiene compliance in all 10 healthcare organizations as well as the implemented (optional) interventions. The compliance at time point 1, the start of the study, was 42.9% (95% CI, 41.4–44.4). After implementing e-learning in 5 organizations and a workshop in 1 organization (between time points 1 and 2), compliance increased by 2.2% to 45.1% (95% CI, 43.4–46.8), but it decreased to 41.2% (95% CI, 39.7–42.7) at time point 3. During this period, 3 organizations implemented a workshop. Hand hygiene compliance increased to 53.9% (95% CI, 52.3–55.5) after the implementation of observer training (in 7 organizations) and team training (in 2 organizations) between time points 3 and 4. One organization implemented observer training after time point 4. At the end of the study, the mean hand hygiene compliance of all organizations was 51.4% (95% CI, 49.8–53.0), a significant (*P*<.001) increase of 8.5% between time points 1 and 5.

### χ^2^ analyses

Tables 1, 2, and 3 show the χ^2^ analyses and the changes in hand hygiene compliance over time by ward type, type of HCW, and healthcare organization. Table 1 shows significant increases in hand hygiene compliance on 5 of the 9 ward types, but 3 of the 9 ward types (ie, pediatric, neonatal, and mixed) showed a significant decrease between time points 1 and 5. Concerning the type of HCW, only nurses showed a significant improvement of 9.2% for hand hygiene compliance (*P*<.001). Table 3 shows great diversity among organizations in hand hygiene compliance change between time point 1 and time point 5 (ranging from −11.5% to +33.3%).

**Table 1:** Hand Hygiene Compliance Change Over Time per Ward Category

	Time Point 1			Time Point 5				
Ward	Correct Opportunities	Total Opportunities	% Compliance	Correct Opportunities	Total Opportunities	% Compliance	*P* Value of Change	% Change
Internal ward	590	1,482	**39.8**	678	1,393	**48.7**	<.001^a^	**8.9**
Surgical ward	465	1,097	**42.4**	516	1,046	**49.3**	.001^a^	**6.9**
Intensive care ward	171	433	**39.5**	176	346	**50.9**	.002^a^	**11.4**
Pediatric ward	85	129	**65.9**	48	98	**49.0**	.010^a^	**−16.9**
Neonatal ward	42	53	**79.2**	36	63	**57.1**	.012^a^	**−22.1**
Emergency ward	56	166	**33.7**	85	199	**42.7**	.079	**9.0**
Gynecology-obstetrics	83	222	**37.4**	84	145	**57.9**	<.001^a^	**20.5**
Mixed ward	255	388	**65.7**	253	436	**58.0**	.023^a^	**−7.7**
Other wards^b^	93	315	**29.5**	246	405	**60.7**	<.001^a^	**31.2**

a Significant if *P*<.05 in χ^2^ analysis.

b Outpatient clinic and cardiology, cardiac catheterization, and ENT wards.

**Table 2:** Hand Hygiene Compliance Change Over Time per Type of Healthcare Worker

	Time Point 1			Time Point 5				
Type of HCW	Correct Opportunities	Total Opportunities	% Compliance	Correct Opportunities	Total Opportunities	% Compliance	*P* Value of Change	% Change
Physicians	183	357	**51.3**	180	380	**47.4**	.291	**−3.9**
Nurses	1,611	3,786	**42.6**	1,936	3,739	**51.8**	<.001^a^	**9.2**
Other HCWs^b^	46	142	**32.4**	6	12	**50.0**	.222	**17.6**

Note. HCW, healthcare worker.

a Significant if *P*<.05 in χ^2^ analysis.

b Nutritionists, physiotherapists, laboratory staff, etc.

**Table 3:** Hand Hygiene Compliance Change Over Time Per Healthcare

	Time Point 1			Time Point 5				
Organization	Correct Opportunities	Total Opportunities	% Compliance	Correct Opportunities	Total Opportunities	% Compliance	*P* Value of Change	% Change
Healthcare organization 1	913	1,909	**47.8**	808	1,368	**59.1**	<.001^a^	**11.2**
Healthcare organization 2	50	101	**49.5**	81	213	**38.0**	.054	**−11.5**
Healthcare organization 3	104	242	**43.0**	112	228	**49.1**	.181	**6.1**
Healthcare organization 4	227	433	**52.4**	189	366	**51.6**	.825	**−0.8**
Healthcare organization 5	182	410	**44.4**	171	437	**39.1**	.121	**−5.3**
Healthcare organization 6	47	76	**61.8**	164	221	**74.2**	.040^a^	**12.4**
Healthcare organization 7	14	65	**21.5**	51	93	**54.8**	<.001^a^	**33.3**
Healthcare organization 8	123	541	**22.7**	266	606	**43.9**	<.001^a^	**21.2**
Healthcare organization 9	42	107	**39.3**	78	129	**60.5**	.001^a^	**21.2**
Healthcare organization 10	138	401	**34.4**	202	470	**43.0**	.010^a^	**8.6**

a Significant if *P*<.05 in χ^2^ analysis.

### Multilevel logistic regression

Table 4 shows the outcome of univariable and multivariable multilevel logistic regressions. The regression analysis shows that all variables are significant in the univariable model. Therefore, all variables are included in the multivariable model.

**Table 4:** Hand Hygiene Compliance With Multilevel Logistic Regression Analysis

Variable	Hand Hygiene Opportunities, No. (%)	Compliance %	Odds Ratio (95% CI) in Univariable Analysis	Odds Ratio (95% CI) in Multivariable Analysis
**Time point**				
Time point 1	4,285 (21.1)	42.9	1.00	1.00
Time point 2	3,497 (17.2)	45.1	1.13 (1.03–1.24)^a^	1.12 (1.02–1.23)^a^
Time point 3	4,432 (21.8)	41.2	0.97 (0.89–1.06)	0.96 (0.88–1.05)
Time point 4	3,941 (19.4)	53.9	1.62 (1.48–1.77)^a^	1.61 (1.47–1.76)^a^
Time point 5	4,131 (20.4)	51.4	1.46 (1.34–1.59)^a^	1.44 (1.32–1.57)^a^
**Ward**				
Internal ward	6,845 (33.7)	46.4	1.00	1.00
Surgical ward	4,977 (24.5)	46.7	1.00 (0.93–1.08)	1.00 (0.93–1.08)
Intensive care ward	1,939 (9.6)	41.9	0.87 (0.78–0.96)^a^	0.88 (0.79–0.97)^a^
Pediatric ward	543 (2.7)	57.8	1.64 (1.37–1.97)^a^	1.69 (1.41–2.02)^a^
Neonatal ward	266 (1.3)	72.6	4.02 (3.04–5.32)^a^	3.96 (2.99–5.25)^a^
Emergency ward	970 (4.8)	35.4	0.60 (0.52–0.69)^a^	0.58 (0.50–0.67)^a^
Gynecology–obstetrics	922 (4.5)	42.0	0.81 (0.70–0.94)^a^	0.83 (0.72–0.96)^a^
Mixed ward	1,901 (9.4)	54.4	1.30 (1.16–1.45)^a^	1.29 (1.15–1.44)^a^
Other ward^b^	1,923 (9.5)	47.5	0.96 (0.85–1.07)	0.95 (0.85–1.07)
**Type of HCW**				
Physicians	2,197 (10.8)	45.8	1.00	1.00
Nurses	17,910 (88.3)	47.0	1.11 (1.02–1.22)^a^	1.05 (0.95–1.15)
Other HCW^c^	179 (0.9)	33.5	0.57 (0.41–0.79)^a^	0.62 (0.45–0.86)^a^

Note. HCW, healthcare worker.

a Significant if *P*<.20 in univariable analysis and if *P*<.05 in multivariable analysis.

b Outpatient clinic and cardiology, cardiac catheterization, and ENT wards.

c Nutritionists, physiotherapists, laboratory staff, etc.

The multivariable model shows that for the variable ‘ward,’ the neonatal ward has highest odds of being compliant in performing hand hygiene (OR, 3.96; 95% CI, 3.99–5.25) compared to the internal ward. This result contrasts with the emergency ward, which had the lowest odds of being compliant (OR, 0.58; 95% CI, 0.50–0.67). Furthermore, a significant increase in hand hygiene compliance was observed for almost all time points, with time point 4 (OR, 1.61; 95% CI, 1.47–1.76) and 5 (OR, 1.44; 95% CI, 1.32–1.57) showing the highest increase. For the last variable, type of HCW, only ‘other HCWs’ shows a significant difference in the multivariable analysis (OR, 0.62; 95% CI, 0.45–0.86) compared to physicians.

## Discussion

The main aim of this study was to investigate the effects of a multimodal, friendly competition intervention on hand hygiene compliance. The core element of the intervention program was the monitoring and feedback of hand hygiene compliance in an existing collaboration of 10 healthcare organizations. Hand hygiene was observed during a period of 2 years at 6-month intervals, following the WHO Five Moments of Hand Hygiene. The results of this study suggest that implementation of a multimodal intervention program framed within a friendly competition setting can increase hand hygiene compliance in healthcare organizations. Comparing time point 1 with time point 5, an overall increase of 8.5% in hand hygiene compliance occurred across all healthcare organizations, with large variations among the organizations (−11.5% to +33.3%). Hand hygiene compliance also differed between ward type and type of HCW, resulting in a low overall increase in compliance on the surgical wards (6.9%) but a much greater increase on the gynecology-obstetric wards (20.5%). The neonatal ward in the current study showed the highest odds (OR, 3.96; 95% CI, 2.99–5.25) of being compliant in the multivariable analysis. At the same time, hand hygiene compliance in the neonatal ward decreased significantly by 22.1% between time points 1 and 5. This finding can be explained by the fact that hand hygiene compliance rates between time points 1 and 5 are very different, but the average hand hygiene compliance of all the 5 time points together is still high.

These findings are in line with other studies in the literature. In a review, Gould et al^18^ and Luangasanatip et al^25^ showed that hand hygiene compliance increased and HAI decreased with different types of interventions. Furthermore, performance feedback was associated with increased hand hygiene compliance. Concerning the wards, Fuller et al^26^ showed an increase in compliance of 10% (*P*<.01) on intensive care wards in a feedback intervention trial with baseline compliance of 70%.^26^ This finding is in line with the increase of 11.4% on the intensive care ward in our present study.

Other literature suggests that nurses usually have a higher compliance rate than physicians.^10^ Moro et al^27^ investigated hand hygiene compliance between nurses, physicians, auxiliary staff, and other HCWs. They studied the effect of a national multimodal “Clean Care is Safer Care” campaign for hand hygiene in 65 hospitals in Italy. The hand hygiene compliance of nurses increased by 25% compared to an increase of 16% among physicians.^27^ In the present study, nurses showed greater improvement in hand hygiene compliance than physicians. The hand hygiene compliance of nurses increased significantly by 9.2% (*P*<.001) between time points 1 and 5. This finding contrasts with the compliance rate of physicians, which showed no significant change in the same period. Possibly, nurses were targeted more specifically by the interventions executed in their organization, for example, through team training on the ward.

This study has a number of strengths and limitations. First, organizations were able to choose the intervention they wanted to implement. In addition to monitoring and feedback of achievements at 6-month intervals, they also chose their own interventions outside the study setting. Therefore, the results reflect the effects of usual improvement activities together with the effect of being part of a study. Although this might make the data less compelling from a scientific point of view, the conclusion remains that improvement is achievable for hospitals implementing interventions on their own, which is a major strength of this study. Another strength is the fact that the hand hygiene observations were not a burden to the HCWs. The HCW participants followed their normal work routines and were observed by unobtrusive observers without interrupting the care processes. All observers followed HCWs during their tasks, (and after consent from the patient) even behind curtains and during washing/showering, etc. This method yielded a true cross section of hand hygiene opportunities without bias to specific opportunities (eg, before or after care). Furthermore, the number and variation in type (eg, academic teaching, rural, revalidation) of participating healthcare organizations and HCWs, as well as hand hygiene opportunities, provides a good representation of healthcare organizations in general. Additionally, the same method is used at all time points, which resulted in a constant number of observations of hand hygiene opportunities over time, which led to less fluctuation in our results.

The first limitation of this study is the uncontrolled, nonrandomized design, which makes it impossible to determine a causal relation between the interventions of “Roll Up Your Sleeves” and hand hygiene compliance. On the other hand, these results provide a better estimate of how hand hygiene could be improved in daily practice outside of a strict study setting. The healthcare organizations were allowed to use other interventions in addition to the interventions offered by “Roll Up Your Sleeves” due to the observational nature of the study. However, it was difficult to monitor the extent of these other interventions, which makes it difficult to estimate how many HCWs were exposed to the interventions within each organization. Another limitation is the long time interval between feedback reports, which might have limited the impact of the intervention. However, due to the continuous attention for hand hygiene compliance between the reports, we expect that this effect was minimal. Furthermore, although the multilevel analyses corrected the effect of the intervention for the type of wards within organizations, other variables, such as hospital size, might have influenced the hand hygiene compliance of HCWs. Lastly, although directly observing hand hygiene behavior is considered the gold standard, it can change the behavior of those being observed (ie, the Hawthorne effect), which could lead to socially desirable behavior (ie, better hand hygiene) or altered behavior. Better hand hygiene behavior only occurs if the observed HCW is aware of the correct opportunities, which would then lead to an overestimation of hand hygiene compliance.^28^ To decrease the possibility of a Hawthorne effect, the observers mentioned to the HCWs that they were observing patient safety in general instead of hand hygiene compliance specifically. Even though the Hawthorne effect may have contributed to an increase in hand hygiene compliance, the increase is mostly attributable to the 5 rounds of observations and the successful interventions of the “Roll Up Your Sleeves” project.

Our results show that the multimodal intervention program of “Roll Up Your Sleeves” in a friendly competition setting was accompanied by a significant overall increase of hand hygiene compliance between the start and the end of the program. Future research is needed to investigate the long-term effects of the intervention program and how the element of competition could be further applied to promote hand hygiene compliance. It is essential to collect data on exposure to the intervention components to understand the effects. Future research could focus more on behavioral and environmental factors that might influence hand hygiene compliance such as access and availability of ABHR on a ward. In Dutch hospitals, the presence of AHBR is highly recommended (and checked) in organizations. However, the influence of location of ABHR on hand hygiene compliance could also be examined. These insights can be applied to further improving hand hygiene compliance, and ultimately, decreasing HAIs.
